# Enhanced Oral Bioavailability of Celecoxib Nanocrystalline Solid Dispersion based on Wet Media Milling Technique: Formulation, Optimization and In Vitro/In Vivo Evaluation

**DOI:** 10.3390/pharmaceutics11070328

**Published:** 2019-07-11

**Authors:** Zhuang Ding, Lili Wang, Yangyang Xing, Yanna Zhao, Zhengping Wang, Jun Han

**Affiliations:** Institute of Biopharmaceutical Research, Liaocheng University, No.1, Hunan Road, Liaocheng 252059, China; dingzhuang@lcu.edu.cn (Z.D.); liliwangmail@163.com (L.W.); kahunsha@163.com (Y.X.); zhaoyanna@lcu.edu.cn (Y.Z.); bioactiveschina@163.com (Z.W.)

**Keywords:** nanocrystal, celecoxib, bioavailability, experimental design, wet media milling, lyophilization

## Abstract

Celecoxib (CLX), a selective COX-2 inhibitor, is a biopharmaceutics classification system (BCS) class II drug with its bioavailability being limited by thepoor aqueoussolubility. The purpose of this study was to develop and optimize CLX nanocrystalline(CLX-NC) solid dispersion prepared by the wet medium millingtechnique combined with lyophilizationto enhance oral bioavailability. In formulation screening, the resulting CLX-NC usingpolyvinylpyrrolidone (PVP) VA64 and sodiumdodecyl sulfate (SDS) as combined stabilizers showed the minimum particle size and a satisfactory stability. The formulation and preparation processwere further optimized by central composite experimentaldesign with PVP VA64 concentration (X_1_), SDS concentration (X_2_) and milling times (X_3_) as independent factors and particle size (Y_1_), polydispersity index (PDI, Y_2_) and zeta potential (Y_3_) as response variables. The optimal condition was determined as a combination of 0.75% PVP VA64, 0.11% SDS with milling for 90 min.The particle size, PDI and zeta potential of optimized CLX-NC were found to be 152.4 ± 1.4 nm, 0.191 ± 0.012 and −34.4 ± 0.6 mV, respectively. The optimized formulation showed homogeneous rod-like morphology as observed by scanning electron microscopy and was in a crystalline state as determined by differential scanning calorimetry and powder X-ray diffraction. In a storage stability study, optimized CLX-NC exhibited an excellent physical stability during six months’ storage at both the refrigeration and room conditions. In vivo pharmacokinetic research in Sprague-Dawley ratsdisplayed that *C*_max_ and AUC_0–∞_ of CLX-NC were increased by 2.9 and 3.1 fold, compared with physical mixture. In this study, the screening and optimizing strategy of CLX-NC formulation represents a commercially viable approach forenhancing the oral bioavailability of CLX.

## 1. Introduction

The large number of drug molecules arising from high-throughput screening have higher lipophilicity and higher molecular weight in the quest for better biological selectivity and specificity with target receptors [1]. These physicochemical properties often lead to a poor water solubility and low dissolution rate of these compounds. At present nearly 40% of chemical drugs being developed at a multitude of pharmaceutical companies suffer from low solubility, potentially resulting in low and erratic oral bioavailability [2,3]. Numerous approaches have thus been developed to improve the solubility and dissolution rate of these poorly soluble drugs, including use of cosolvents, salt or prodrug formation, lipid-based formulations, complexation with cyclodextrins, amorphization, microemulsions and nanotechnology [4].

Among the above approaches, the nanocrystals technique has become a promising approach [4]. This technique provides the fastest breakthrough from design development to commercial production, whereby the first product based on nanocrystals entered the pharmaceutical market only 10 years after the first patent application, in contrast to the 25 years of the liposomes technique [5]. Nanocrystals are nanosized crystals of drug particles with the size lower than 1 µm stabilized by surface stabilizers [6,7], and are characterized by higher solubility and faster dissolution rate due to their reduced size and increased surface area [8,9]. Several methods have been used for preparing drug nanocrystals, and fallen into two categories: bottom up and top down method, according to the route of drug nanoparticle formation. Although easily performed at a laboratory scale, a bottom up method, such as anti-solvent and evaporative precipitation, seems to be unavailable to industrial production due to numerous limitations from difficult crystal control and potential organic solvent residues. Thus, the top down method has become the first choice for commercial production of drug nanocrystals [10,11]. The wet media milling technique, as an effective top down method for producing drug nanocrystals, exhibits the advantages of high drug loading, high production efficiency, short development cycle and flexibility for industrialization [12,13]. Actually, several nanocrystals formulations prepared by wet media milling have been operating in the market in recent years, exemplified by Emend^®^ (aprepitant, Merck, USA), Invega^®^ (paliperidone, Janssen, Belgium), and Rapamune^®^ (sirolimus, Pfizer/Wyeth, USA), Ritalin LA^®^ (methylphenidate HCl, Novartis, Switzerland) and Tricor^®^ (fenofibrate, AbbVie, USA) [10,14].

In the wet media milling process, coarse drug particles suffer collision and impaction due to the moving of milling beads, resulting in a rapid decrease of drug particle size. In addition, the application of an optimal stabilizer in milling system is necessary to provide an adequate storage stability. Thus, it is well established that drug particle nanocrystallization by wet media milling is influenced by numerous formulations and process parameters, such as properties and concentration of drug and stabilizers, loading and size of milling beads, milling times, and stirrer/agitation speed [6,12]. Before mass production, it is very important and necessary to optimize these formulations and process parameters. However, optimization by a traditional screening approach is time-consuming and does not reflect the complex interaction of formulation and process parameters [15]. Thus, the quality by besign (QbD) concept is strongly recommended to be applied in the development of pharmaceuticals. QbD approach for the production of drug nanocrystals can be divided into three steps: (i) selection of excipients and production method, (ii) establishment of critical quality attributes (CQAs), such as particle size, zeta potential or solubility, and (iii) constitution of a ‘design space’ by design of experiments (DoE) [16,17]. The QbD approach has been widely used in the formulation design and process optimization of various complex dosage forms. Nevertheless, only a few studies focus on the optimization of wet media milling process for the preparation of drug nanocrystals using systematic experimental design method [18].

Celecoxib (CLX) is a selective cyclooxygenase-2 inhibitor used for the treatment of rheumatoid arthritis, osteoarthritis and management of pain. As a biopharmaceutics classification system (BSC) class II drug, the low aqueous solubility of CLX (3–5 μg/mL) limits its absorption from the gastrointestinal tract and leads to a poor oral bioavailability [19]. Numerous pharmaceutical strategies have been applied to increase the solubility and dissolution rate of CLX, such as complexation with drug carriers [20], solid dispersions [19,21], emulsions [22], micelles [23], suspensions [24,25] and liposomal [26]. In this study, a stable CLX nanocrystalline (CLX-NC) solid dispersion was produced by using wet media milling technique combined with lyophilization. The formulation and preparation process was extensively optimized by employing three factors/five levels rotatable central composite experimental designin order to achieve the lowest size of CLX-NC. The physicochemical characterization of optimized CLX-NC were systematically investigated by scanning electron microscopy (SEM), differential scanning calorimetry (DSC) and powder X-ray diffraction (PXRD). Finally, storage stability, apparent solubility, in vitro dissolution rate, and in vivo oral pharmacokinetics of optimized CLX-NC were performed.

## 2. Materials and Methods

### 2.1. Materials

Celecoxib (CLX), used as micronized crystalline power (diameter, D90: ~130µm), was purchased from Yijing Industrial Co. Ltd. (Shanghai, China). Hydroxypropylmethylcellulose acetate succinate (HPMC-AS, AquaSolve^™^ HG) and hydroxypropylcellulose (HPC, Klucel^™^ EF Pharm) were kindly gifted by Ashland Asia Pacific (Shanghai, China). Polyvinylpyrrolidone—polyvinyl acetate copolymers (PVP VA64, Kollidon^®^ VA 64) and polyvinylpyrrolidone (PVP K30, Kollidon^®^ 30) were generously provided by BASF (China) Ltd. (Shanghai, China). Above these polymers were used singly as primary stabilizer in the preparation of the nanocrystal formulations. Sodiumdodecyl sulfate (SDS), used as secondary stabilizer, was purchased from Hunan Erkang Pharmaceutical Co. Ltd. (Changsha, China). The water used in all experiments was ultrapurified Milli-Q water (Millipore, Billerica, MA, USA). All other chemicals and reagents were of analytical or chromatographic grade.

Male Sprague–Dawley rats (6–8weeks, 200 ± 20 g) were purchased from Pengyue experimental animal breeding co. LTD (Jinan, China). The adjustable feeding for the experimental animals before experiments were conducted according to the procedures described by Zhao et al. [27]. All the procedures of the experimentation were strictly in compliance with the guidelines and policies for Animal Experiments Ethical and Regulatory as approved by the Animal Ethics Committee of Liaocheng University (approval code: 2018-05009), 2018.

### 2.2. Preparation of Celecoxib Nanocrystalline (CLX-NC) and Physical Mixture

CLX nanosuspension was first prepared by a laboratory scale milling apparatus (Dyno^®^-Mill Multi Lab, WAB, Basel, Switzerland). CLX (4.0%, *w*/*v*), primary stabilizer (0.8%, *w*/*v*) and secondary stabilizer (0.1%, *w*/*v*) were dispersed in aqueous solution (400 mL) using a magnetic stirrer operating at 500 r/min. The suspension was poured into a milling chamber (500 mL) loaded with 300 mL of yttrium-stabilized zirconium oxide beads (0.3 mm diameter). The milling operation was performed at a stirrer-tip speed of 10 m/s in a re-circulation mode with the suspension being fed at a rate of 200 mL/min. The temperature of the suspension was controlled at less than 30 °C by circulating cooling water through the milling chamber jacket. The nanosuspension was separated from the beads by a screen sheet with gap width of 0.13 mm. Subsequently, the resulting nanosuspension was dried using a laboratory scale freeze dryer (VirTis BenchTop Pro 8L, SP Industries, Warminster, PA, USA). The lyophilization process was performed for 10 h at −55 °C under a vacuum pressure of 100 mTorr. Finally, the obtained solid powder was further pulverized gently, then sealed in glass vials. The physical mixture (PM) containing CLX and stabilizer was prepared in the same ratio as optimized CLX-NC. All the powders were mixed gently using mortar and pestle until an uniform mixture was obtained. The obtained PM was also stored in sealed glass vials. Each trial was performed in triplicate.

### 2.3. Characterization of CLX-NC

#### 2.3.1. Particle Size and Zeta Potential

The mean particle size and polydispersity index (PDI) of CLX-NC were measured by dynamic light scattering (DLS) technique using a Zetasizer Nano ZSP system (Malvern Instruments, Malvern, UK). Zeta potential value was measured by laser doppler micro-electrophoresis technique using the same instrument. CLX-NC was re-dispersed with deionized water to form nanosuspension and further diluted to achieve a suitable concentration for analysis. Each sample was measured in triplicate.

#### 2.3.2. Scanning Electron Microscopy (SEM)

The morphology of CLX crude powder and optimized CLX-NC was observed using a scanning electron microscope (S-4800, Hitachi Limited., Tokyo, Japan) at 10kV. Each sample was fixed on an aluminium stub using double-side adhesive tape, and sputter coated with gold-palladium to make a thickness of 10 nm before observing.

#### 2.3.3. Differential Scanning Calorimetry (DSC)

DSC thermograms were measured using DiscoveryDSC system (TA Instruments, New Castle, DE, US). An accurate amount (4.0 mg) of each sample was weighed and placed in an aluminium pan with pierced lid. A heating rate was employed in the range of 20–200 °C at 10 °C/min under nitrogen gas with 50 mL/min. DSC was precalibrated for baseline using an empty pan.

#### 2.3.4. Powder X-ray Diffraction Analysis (PXRD)

PXRD patterns were collected using D8 Advance diffractometer (Bruker, Karlsruhe, Germany) with Cu-Ka radiation (1.5406 Å) operated at 36 kV and 20 mA. Data were obtained in the range of 5–50°(2θ) at 1.5°/min with a step of 0.04°.

### 2.4. Experimental Design

The suitable polymer used as primary stabilizer was selected for the preparation of CLX-NC formulation. 0.8 % (*w*/*v*). HPMC AS, HPC, PVP VA64 and PVP K30, were used separately as primary stabilizers. SDS (0.1%, *w*/*v*) as secondary stabilizer was kept constant. After milling and drying process, the particle size, PDI and zeta potential of CLX-NC were determined to evaluate the effects of the used stabilizers. The selection was based on particle size distribution and short-term stability of the resultant CLX-NC. Subsequently, concentrations of the selected stabilizers and milling times were selected as independent factors and further optimized using central composite design (Design-Expert 8.0.6.1 software, Stat-Ease Inc., Minneapolis, MN, USA). In the above studies, concentrations of CLX (4%, *w*/*v*), media size (0.30 mm), and milling speed (10 m/s) were kept constant. Table 1 lists the independent factors along with their levels. Twenty experimental runs were prepared and evaluated extensively by particle size (Y_1_), PDI (Y_2_) and zeta potential (Y_3_) as CQAs (response variables). Design-Expert 8.0.6.1 software was used for data treatment and response surface plots generation.

### 2.5. Storage Stability Study

The physical stability of CLX-NC was evaluated after a short- (25 °C) or long-term (4 °C and 25 °C) storage. The samples stored in the sealed glass vials were periodically withdrawn at 0.5 and 1 months for short-term stability, or 3 and 6 months for long-term stability. Particle size, PDI and zeta potential were determined to evaluate the physical stability.

### 2.6. High-Performance Liquid Chromatography (HPLC)Analysis of CLX

CLX concentration was quantified by a 1525 high-performance liquid chromatography (HPLC) instrument (Waters, Milford, MA, USA). 50 μL of sample was injected into a Eclipse XDB-C18 (5 μm, 4.6 mm × 250 mm, Agilent, Santa Clara, CA, USA) analytical column under 30 °C. Acetonitrile/water as the mobile phase was pumped at a ratio of 60/40 (*v*/*v*) using a flow rate of 1.0 mL/min. The detection wavelength was set at 252 nm. CLX concentration was calculated using a standard curve produced by definite CLX concentrations in acetonitrile. Each sample was measured in triplicate.

### 2.7. Apparent Solubility

The apparent solubility of CLX, PM and CLX-NC was measured in hydrochloric acid solution (pH 1.2), phthalate buffer (pH 4.6), phosphate buffer (pH 6.8) and distilled water using the shake flask method. 5 mg of each sample was added into sealed glass vials with 10 mL of each solvent. These vials were vibrated at 120 r/min for 72 h in 37 ± 0.5 °C. After equilibrium was reached, the sample was withdrawn and centrifuged at 12,000 g for 10 min to remove the undissolved drug. The supernatant was used to determine the content of CLX by HPLC assay. Each sample was measured in triplicate.

### 2.8. In Vitro Dissolution Study

Dissolution studies of CLX crude powder, PM and CLX-NC were performed in Distek 7100 automated dissolution test apparatus (Distek Inc., North Brunswick, NJ, USA). The temperature and paddle speed were set at 37 °C and 100 rpm, respectively. The dissolution media included hydrochloric acid solution (pH 1.2), phthalate buffer (pH 4.6), phosphate buffer (pH 6.8) and distilled water. 0.3% (*w*/*v*) SDS was added into each medium to maintain the sink condition. The samples (equivalent to 50 mg CLX) were added into the vessels containing 900 mL dissolution medium. 2 mL of dissolution medium were withdrawn at the predetermined time intervals of 5, 10, 20, 30, 45, 60 and 120 min, and immediately replaced with equal volume of fresh dissolution medium. The collected sample was centrifuged at 12,000 g for 10 min to remove the undissolved drug. The supernatant was used to determine the content of CLX by HPLC assay. Each sample was measured in triplicate.

### 2.9. In Vivo Oral Bioavailability

Pharmacokinetic study was performed with male Sprague–Dawley rats (weight: 200 ± 20 g). Twelve rats were randomly separated into two groups, PM group (control) and CLX-NC group. The rats had free access to water but fasted in 12 h before the experiment. PM and CLX-NC were administered orally at CLX doses of 50 mg/kg. 1.5 mL of blood sample were collected in heparinized tube from retro orbital plexus at the predetermined time intervals of 0.25, 0.50, 1, 1.5, 2, 3, 4, 6, 8, 12 and 24 h. The plasma was separated by centrifuging the blood sample at 5000 g for 5 min and stored at −20 °C until further analysis. 300 µL of extracting solvent of acetonitrile was added to each 200 µL plasma sample. The mixture was vortexed for 10 min and centrifuged at 12,000g for 10 min. The supernatant was used to determine the content of CLX.

The pharmacokinetic parameters including area under the curve (AUC_0–24h_), elimination half life (*t*_1/2_) were calculated by DAS 2.0 software (SPPS Inc., Chicago, IL, USA). Maximum concentration (*C*_max_) and time to reach maximum concentration (*T*_max_) were obtained from plasma concentration-time curve.

### 2.10. Statistical Analysis

All results were described as mean ± standard deviation (SD) using GraphPad^®^Prism 7.0 (GraphPad software, San Diego, CA, USA). The evaluation of data was performed by the *t*-test and * *p* values < 0.05 were considered as statistically significant.

## 3. Results and Discussions

### 3.1. Screening of Polymer Stabilizers

**Figure 1:**
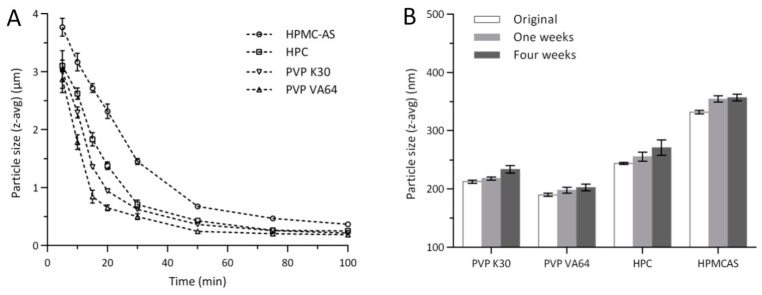
Particle size distributions during (**A**) milling process and (**B**) short-term storage process of celecoxib nanocrystalline (CLX-NC) containing different polymer stabilizers (mean ± SD, n = 3).

The enormous surface area of nanosized drug particles leads to increase in Gibbs-free energy, which is unfavorable to maintain the stability of products. The application of appropriate stabilizers are critical to reduce particle aggregation of nanocrystals during preparation and storage [9]. In this study, the adequate polymeric and ionic surfactants as the stabilizer combination were chosen to maintain the stability of CLX-NC. Polymer stabilizers used in CLX-NC formulation were selected by evaluated the particle size distribution and short-term stability of CLX-NC. 0.8% (*w*/*v*) of HPMC-AS, HPC, PVP VA64 and PVP K30 as primary stabilizers in combination with SDS (0.1 %, *w*/*v*) as the ionic stabilizer were evaluated. Figure 1 shows that the particle size distributions during milling and short-term storage process of CLX-NC containing different polymer stabilizers. The results show all polymer stabilizers combined with SDS could reduce and maintain the nanosized particles of CLX-NC effectively. In the initial 30 min of milling process, PVP K30 and PVP VA64 provided better milling efficiency compared with HPC, due to lower viscosity of PVP K30 and PVP VA64 in the same concentration (Figure S1). Although possessing low viscosity, HPMC-AS still gave the worst performance on milling efficiency. This might be attributed to an excess of HPMC-AS leaded to the self-aggregation because of its relative lower water solubility, and thus reduced the apparent breakage efficiency [28]. In this screening, the particle sizes of CLX-NC with different polymer stabilizers were in the order of HPMC-AS > HPC > PVP K30 > PVP VA64. The particle size of CLX-NC after short-term storage in 25 °C showed no significant particle growth and agglomeration. Due to the minimum particle size distribution in the milling and storage process, the combination of PVP VA64 and SDS was selected for further optimization of the CLX-NC formulation.

### 3.2. Optimization of CLX-NC Using Central Composite Design

The application of stabilizers in a nanocrystals formulation is known to be indispensable for facilitating drug particle breakage and maintaining system stabilization, while the amount of stabilizers added exhibits a subtler influence on product quality ofnanocrystals by milling. Lower concentration of stabilizers is inadequate to keep system stabilization, while higher concentration of stabilizers is detrimental for nanocrystals formation and stability due to higher viscosity and solubilization [12]. Simultaneously, the combinations of different stabilizers, such as polymers and ionic surfactants, are found to have a synergistic effect in nanocrystals stabilization [18]. Among process parameters, milling time is considered a very important one, which is conducted to reduce drug particle size and improve homogeneity. However, excessively long milling times can lead to the destabilization of nanocrystals system due to heat generation caused by energy accumulation. Furthermore, the desired milling times, to some extend, depends on stabilizers concentration due to viscous dampening. In the preliminary assessment for preparing CLX-NC, above three factors also exhibited a significant influence on the actual milling result of CLX-NC (Figure S2, Table S1). Thus, the concentrations of PVP VA64 and SDS, as well as milling times were selected as the three critical factors for optimization by central composite experiment design. 20 experimental runs were conducted in this study. The results of the selected responses such as particle size, PDI, and zeta potential for all experiments are summarized in Table 2.

PDI: polydispersity index.Model selection for response analysis was performed, and the quadratic model was found to be the best description of the relationship among PVP VA64 concentration (X_1_), SDS concentration (X_2_) and milling times (X_3_) as independent factors and CLX-NC particle size (Y_1_), PDI (Y_2_) and zeta potential (Y_3_) as response variables. The fit summary for each response is listed in Table S2. After multiple linear regression analysis of the data, the following polynomial equations describing the quantitative effect of studied independent factors and their interactions on the responses were generated:

Particle Size (Y_1_) = 157.38 − 5.64 × X_1_ + 5.18 × X_2_ − 4.65 × X_3_ + 13.80 × X_1_X_2_ + 3.68 × X_1_X_3_ − 1.05 × X_2_X_3_ + 2.79 × X_1_^2^ +71.72 × X_2_^2^ + 5.05 × X_3_^2^ (1)

PDI (Y_2_) = 0.21 + 0.020 × X_1_ − 0.043 × X_2_ − 0.017 × X_3_ − 0.024 × X_1_X_2_ − 0.004 × X_1_X_3_ + 0.004 × X_2_X_3_ + 0.007 × X_1_^2^ + 0.025× X_2_^2^ + 0.005 × X_3_^2^ (2)

Zeta Potential (Y_3_) = −34.59 + 2.99 × X_1_ − 4.52 × X_2_ + 0.51 × X_3_ + 1.64 × X_1_X_2_ + 2.11 × X_1_X_3_ + 1.36 × X_2_X_3_ + 3.42 × X_1_^2^ +3.40 × X_2_^2^ + 0.42 × X_3_^2^ (3)

In ananalysis of variance (ANOVA, Table 3), SDS concentration (X_2_^2^) was found to be the most influential factor on the particle size of CLX-NC. Perturbation graphs (Figure S3A) showed that a sharp reduction of CLX-NC size occurred in the low concentration of SDS (0.05 to 0.09%, *w*/*v*); however, the particle size of CLX-NC was increased rapidly following a further addition of SDS. The second-order interaction of combinational stabilizers concentrations (X_1_X_2_) also exhibited a significant effect on CLX-NC particle size. SDS concentration (X_2_) was also a major influencing factor on PDI of CLX-NC. Meanwhile, effects of the other two factors (X_1_ and X_3_) and the second-order interaction of combinational stabilizers concentrations (X_1_X_2_) were also statistically significant. In this experimental design, milling times in the range of 50 to 100 min was not a key factor affecting the particle size and PDI due to relative low viscosity and desired thermal control of milled suspensions. Zeta potential of CLX-NC formulation was also influenced by the stabilizers concentrations (X_1_, X_2_, X_1_^2^ and X_2_^2^). Furthermore, more interactions (X_1_X_2_, X_1_X_3_ and X_2_X_3_) between different factors were also statistically significant on zeta potential of the developed formulations. These results are consistent with the perturbation graphs (Figure S3).

**Figure 2:**
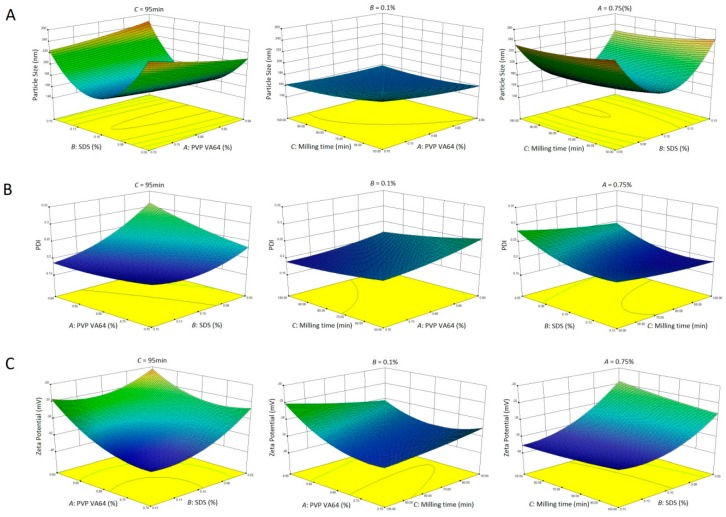
Response surface profiles showed the effects of polymer concentration (X_1_), surfactant concentration (X_2_) and the milling times (X_3_) on (**A**) particle size,(**B**) polydispersity index (PDI) and (**C**) zeta potential of CLX-NC.

The three-dimension response surface plots were used for better evaluation of the factor effects on the CQAs. Figure 2 presents the effect of the interactions between different formulation and process factors on particle size (Y_1_, Figure 2A), PDI (Y_2_, Figure 2B), and zeta potential (Y_3_, Figure 2C) of CLX-NC. In case of Y_1_, response surface plot (Figure 2A) showed that when low SDS concentration was used for milling, the increase of amounts of PVP VA64 could lead to more efficient particle size reduction, probably because more supplement of polymer stabilizer was necessary to maintain the desired stabilization in the absence of ionic stabilizer. However, when high SDS concentration was used, more polymers caused the slight growth of CLX-NC particle size. It could be attributable to the increased viscosity of the suspension caused by additional polymers. Response surface plots of response Y_1_ and Y_2_ exhibited a relative high similarity. In Figure 2B, it was clearly revealed that more narrow particle size distribution was achieved with more milling times and higher ionic stabilizer concentration. In case of Y_3_, Figure 2C exhibited that zeta potential of CLX-NC formulation was decreased on increasing ionic stabilizer concentration. In contrast, a rising trend of zeta potential was observed on increasing polymer concentration. This phenomenon has been reported by Ahuja et al., and has beenattributed to the increased adsorption of polymer onto drug surface resulted in a decrease of absolute value of zeta potential [29].

### 3.3. Model Validation

The main purpose of this research is to identify the design space where all CQAs are desired. In our present study, the desired CQAs were set as <200 nm particle size, <0.2 PDI, and <−25 mV zeta potential due to better dissolution and stability of drug nanocrystals [12,13]. Design space depicted by the yellow overlap region is shown in Figure S4. The desirability function was further evaluated to acquire the best formulation for the lowest particle size of CLX-NC in the design space. This optimal condition was determined as follows: 0.75% PVP VA64, 0.11% SDS with milling for 90 min. Furthermore, the desirability of the model was validated by three checkpoint experiments, including the optimal formulation (Table 4). The verification results of the optimal formulation showed the predicted values of the particle size, PDI and zeta potential of CLX-NC were 159.3 nm, 0.194 and −35.8 mV, respectively; while the measured values were 152.4 ± 1.4 nm, 0.191 ± 0.012 and −34.4 ± 0.6 mV, respectively. It can be seen that the deviations between the measured and predicted values of three checkpoints were less than 5%, which showed that the equation fits well with the actual situation and the optimization results were reliable.

### 3.4. Quality Evaluation of CLX-NC

#### 3.4.1. Particle Size and Zeta Potential Analysis

**Figure 3:**
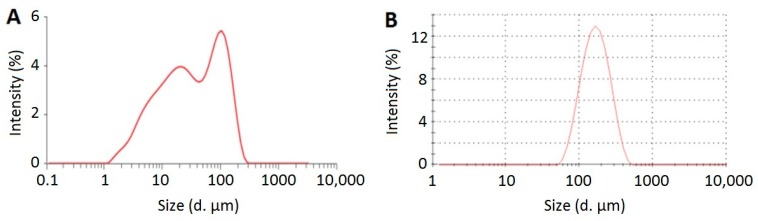
Particle size distributions of (**A**) CLX crude powder and (**B**) optimized CLX-NC.

The particle size distributions of CLX crude powder and optimized CLX-NC are presented in Figure 3. As shown in Figure 3, optimized CLX-NC showed a mean particle size (152.4 ± 1.4 nm) approximately three orders of magnitude lower than CLX crude powder (31.1 ± 4.5 μm). The significant reduction of particle size is favorable for enhancing the dissolution rate and oral bioavailability. Optimized CLX-NC also showed a narrow size distribution with a PDI value of 0.191 ± 0.012, which could avoids problems with the variable solubility of drug particles in different sizes, thus restraining Ostwald ripening and providing long-term stability [30]. In addition, a zeta potential of −34.4 ± 0.6 mV was observed in optimized CLX-NC. The lower zeta potential could provide electrostatic repulsion to prevent from aggregation and agglomeration of drug nanoparticles.

#### 3.4.2. Morphology Evaluation

**Figure 4:**
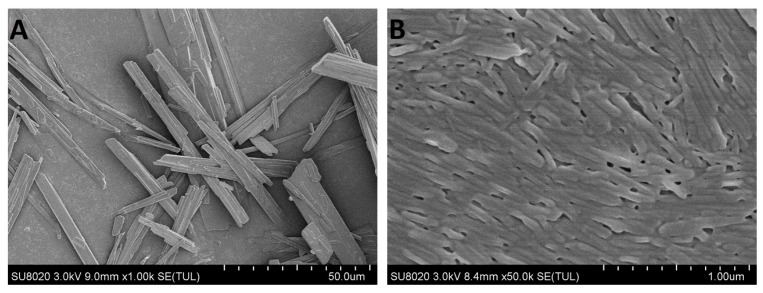
Scanning electron microscope (SEM) images of (**A**) CLX crude powder and (**B**) optimized CLX-NC.

SEM micrographs of CLX crude powder and optimized CLX-NC are shown in Figure 4. It can be seen that CLX cruder powder existed as the predominant long-needle shaped crystals, while optimized CLX-NC obtained by the milling process exhibited a similar shape and more homogeneous crystalline particles with a considerable reduction in size. In addition to particle size analysis by DLS, SEM images provide more evidence that milling process resulted in significant reduction and homogenization for drug particle, which could be benefit to increase oral bioavailability.

#### 3.4.3. Differential Scanning Calorimetry (DSC)Analysis

**Figure 5:**
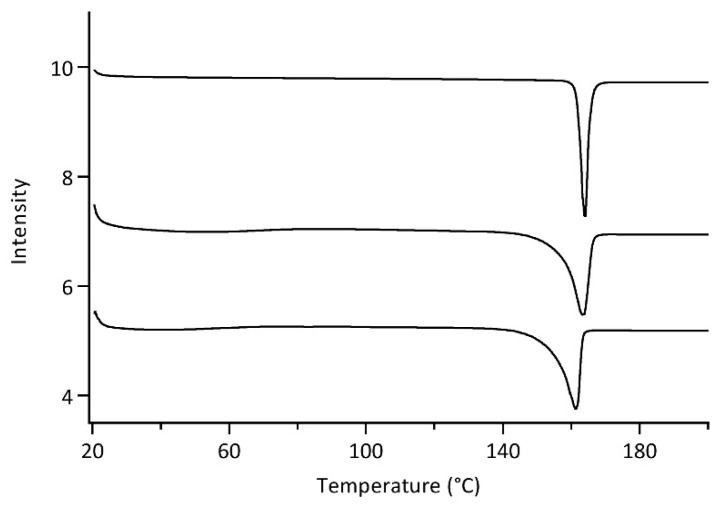
Differential scanning calorimetry (DSC) thermograms of (**A**) CLX crude powder, (**B**) physical mixture (PM) and (**C**) optimized CLX-NC.

DSC thermograms of the CLX crude powder, PM and optimized CLX-NC are shown in Figure 5. CLX crude powder exhibited a sharp endothermic peaks at 164.5 °C associated with the melting point of crystalline CLX [31], while the stabilizers mixture (PVP VA64 and SDS) with the ratio of optimized formulation did not reveal any melting peaks in DSC thermograms due to its amorphous state (data not shown). Compared with CLX crude powder, PM showed a slight early onset of endotherm and 1 °C reduction in the melting point, which could result from the miscibility of the drug with stabilizers. Optimized CLX-NC exhibited a endothermic peaks at 161.4 °C, although the endotherm showed a similar early onset and a further reduction in the melting point, which could be attributed to the reduced particle size to nanometer as per the Gibbs–Thomson equation and the presence of stabilizers [32]. DSC thermograms suggested that the crystalline state of CLX was largely retained and no substantial change in crystalline state transition occurred during preparation process. This conclusion was further indicated by PXRD.

#### 3.4.4. Powder X-ray Diffraction (PXRD)Analysis

**Figure 6:**
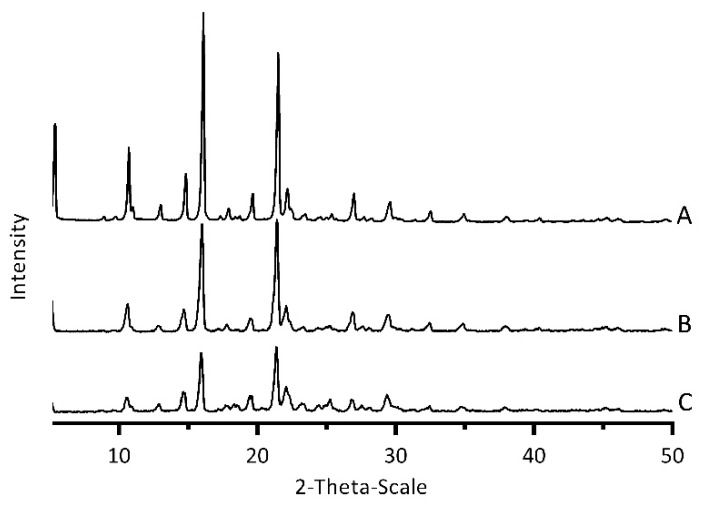
Powder X-ray diffraction (PXRD) patterns of (**A**) CLX crude powder, (**B**) PM and (**C**) optimized CLX-NC.

In order to further confirm the crystalline state of CLX-NC, PXRD analyses of CLX crude powder, PM and optimized CLX-NC were performed (Figure 6). CLX exhibited sharp, diagnostic peaks in the region 5–30° of 2θ values associated with the crystalline state of CLX, while the stabilizers mixture (PVP VA64 and SDS) showed no obvious peak in the diffraction pattern (data not shown). Similar diffraction patterns were obtained for the samples of PM and optimized CLX-NC. However, the peak intensities of PM and optimized CLX-NC showed a slight reduction, which probably be attributed to drug particle size reduction and the influence of stabilizers [29]. Several drug nanoparticle manufacturing techniques, such as antisolvent precipitation, spray drying, high pressure homogenization and media milling, tend to create partial amorphization and crystalline transformation [13]. Although amorphous drug can be benefit to improve dissolution rate and oral bioavailability, the instability of amorphous particles also brings risks to drug use after long-term storage. Thus, retaining crystalline state of bulk drug is important during production process. In this study, the above results concluded that the optimized preparation process did not induce significant amorphous or polymorphic transitions of CLX-NC.

#### 3.4.5. Apparent Solubility Determination

**Figure 7:**
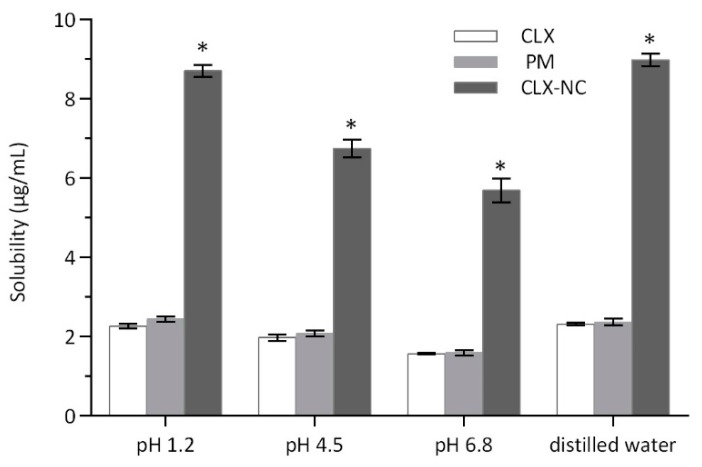
Apparent solubility of CLX crude powder, PM and optimized CLX-NC in different dissolution media (mean ± SD, n = 3). * implies significant at p ≤ 0.05 compared to CLX crude powder.

Figure 7 displays the apparent solubility of CLX crude powder, PM and optimized CLX-NC in hydrochloric acid (HCl) solution (pH 1.2), phthalate buffer (pH 4.5), phosphate buffer (pH 6.8) and distilled water. The apparent solubility of optimized CLX-NC was significantly increased, approximately 4.0 fold (8.90 ± 0.15 μg/mL vs. 2.25 ± 0.06 μg/mL) in pH 1.2 solution, over 3.4 fold (6.74 ± 0.23 μg/mL vs. 1.97 ± 0.08 μg/mL) in pH 4.5 buffer and over 3.6 fold (5.69 ± 0.30 μg/mL vs. 1.57 ± 0.02 μg/mL) in pH 6.8 buffer when compared with CLX crude powder. The Ostwald–Freundlich equation provides an explanation on the increase in apparent solubility of CLX-NC: As the particle decreases, there is an increase in dissolution pressure due to strong curvature of nanoparticles [11]. In addition, the apparent solubility of PM indicates that the solubilization caused by the stabilizers in formulation was negligible (2.44 ± 0.07 μg/mL in pH 1.2 solution, 2.07 ± 0.07 μg/mL in pH 4.5 buffer and 1.58 ± 0.07 μg/mL in pH 6.0 buffer).

#### 3.4.6. In Vitro Drug Release Study

**Figure 8:**
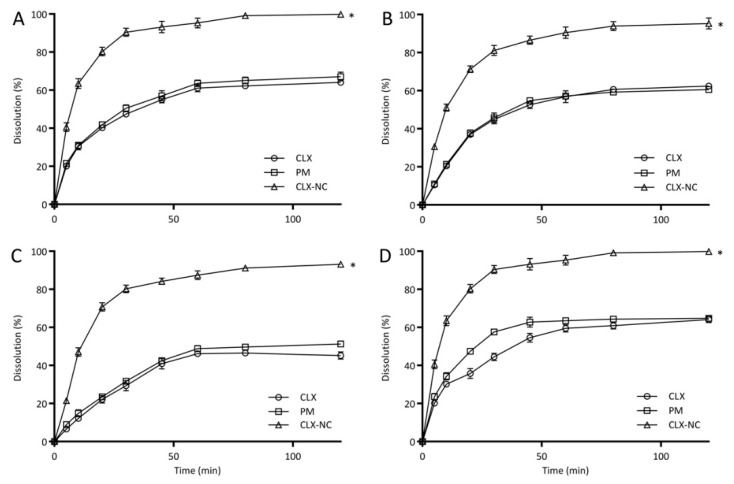
In vitro dissolutionprofiles of CLX crude powder, PM and optimized CLX-NC in different dissolution media (**A**: pH 1.2, **B**: pH 4.5, **C**: pH 6.8 and **D**: distilled water) (mean ± standard deviation (SD), n = 3). * implies significant at p≤0.05 compared to CLX crude powder.

Figure 8 shows the dissolution profiles of CLX crude powder, PM and optimized CLX-NC in four different buffers containing 0.3% SDS over a time period of 120 min. In pH 1.2 solution, optimized CLX-NC displayed a significant enhancement in dissolution rate (95.3 ± 2.5%) in 60 min compared with the CLX (61.1 ± 1.9%) and PM (63.6 ± 1.7%). Similarly, the dissolution rate of optimized CLX-NC was improved in pH 4.5 buffer (90.5 ± 3.0% vs. 56.8 ± 3.0%, 57.2 ± 1.0%) and pH 6.8 buffer (87.4 ± 2.2% vs. 46.2 ± 0.9%, 48.9 ± 1.6%) in 60 min, respectively. Moreover, CLX-NC exhibited markedly increased dissolution velocities (>50% of dissolution rates in 10 min) in all different buffers compared with the CLX and PM (<29.1% and <30.5%, respectively). Increase in dissolution rate of optimized CLX-NC can be explained by the Noyes–Whitney equation, where the enhanced dissolution rate can be attributed to the decreased particle size of drug resulting in enhancement in surface area and decrease in the diffusion layer thickness available for dissolution [8]. In addition, PM exhibited similar dissolution behavior with the CLX, indicating that the stabilizers in formulation had no discernibleeffect on the dissolution rate of CLX.

#### 3.4.7. Storage Stability Study

**Figure 9:**
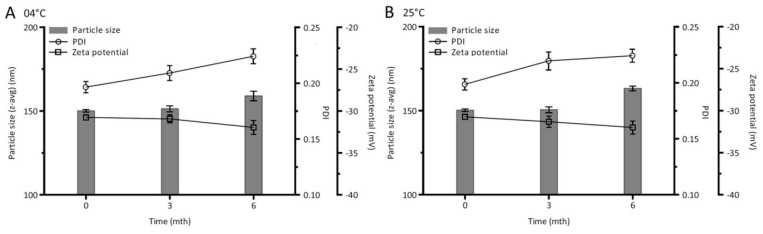
Particle size, PDI and zeta potential of optimized CLX-NC during six months of storage at different temperatures (**A**: 4 °C and **B**: 25 °C) (mean ± SD, n = 3).

In order to evaluate the physical stability, the performance parameters such as particle size, PDI, and zeta potential of optimized CLX-NC during storage for six months at 4 °C and 25 °C were detected and are shown in Figure 9. The particle size of optimized CLX-NC was found to be stable at end of three months and appeared a slight increment after six months at both temperatures. However, the increment was statistically insignificant (p>0.05) when compared to those at an initial time. In spite of exhibiting continuous increase, the PDI of optimized CLX-NC during six months’ storage still retained below 0.25, indicating a narrow particle size distribution [33]. The particle size distributionof CLX-NC shown in Figure S5indicated that there is still one peak and no secondary peaks were observed aftersix months’ storage. Furthermore, the absolute zeta potential values during storage at both temperatures remained higher than 30, which was important to prevent aggregation of drug particles by electrostatic effect [34]. These results suggested optimized CLX-NC had a remarkable stability. This stability should be attributed to the presence of PVP VA64 and SDS, which served as an inhibitor of drug particle growth by adsorbing onto the surface of the drug nanoparticles.

#### 3.4.8. In Vivo Oral Bioavailability

**Figure 10:**
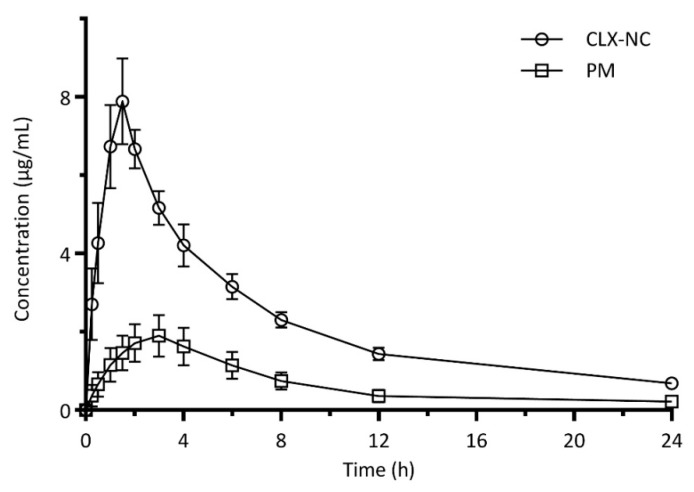
Plasma concentration-time curves of optimized CLX-NC and physical mixture (PM) in rats after oral administration(mean ± SD, n = 6).

The plasma concentration-time curves of optimized CLX-NC and PM in rats after oral administration are presented in Figure 10, and the associated pharmacokinetic parameters are summarized in Table 5. In comparison with PM, nanocrystals’ formulation exhibited significantly enhancement in the oral bioavailability, which was clearly revealed by the increased *C*_max_ (7.88 ± 0.72 μg/mL vs. 2.70 ± 0.25 μg/mL) and AUC_0–∞_ (66.75 ± 2.51 μg·h/mL vs. 21.53 ± 3.02 μg·h/mL) values. In addition, CLX-NC showed faster *T*max (1.50 ± 0.32h vs. 2.83 ± 0.41h), indicating a more rapid absorption rate and higher absorption amount. The enhanced oral bioavailability of CLX-NC could be attributed to the increased apparent solubility and dissolution rate as determined by the aforementioned in vitro studies. Additionally, drug nanocrystals are also known to exhibit greater mucosal adhesion to the gastrointestinal tract, which can prolong gastrointestinal transit time to improve oral bioavailability [3,35]. Furthermore, other mechanisms such as the uptake of drug nanocrystals by M cells may also result in the improved oral bioavailability of CLX-NC [36].

## 4. Conclusions

Nanocrystallization presents a promising strategy to increase the apparent solubility, dissolution rate and oral bioavailability of hydrophobic drugs. The present research investigated the suitability of nanocrystalline solid dispersion prepared by the wet medium milling technique combined with lyophilization for the enhancement of the oral bioavailability of CLX. CLX-NC was successfully prepared using the chosen PVP VA64 and SDS as the combined stabilizers, and further optimized using a central composite design. The optimized formulation was found to have an adequate particle distribution and no substantial crystalline change occurred after the milling process. Quality evaluation indicated that CLX-NC could provide excellent physical stability during six months’ storage at both the refrigeration and room conditions. Furthermore, in vivo pharmacokinetic study of CLX-NC in rats demonstrated a significant improvement in oral bioavailability. The screening and optimizing strategy of CLX-NC formulation in the study represents a commercially viable approach to improve the oral bioavailability of CLX.

## Figures and Tables

**Table 1:** Independent factors and their levels in central composite design.

Independent Factors		Design Level	
Coded	Actual Parameters	Coded Value	Actual Value
X_1_	Concentration of polymer stabilizer (% *w*/*v*)	−1.68	0.63
		−1	0.7
		0	0.8
		+1	0.9
		+1.68	0.97
X_2_	Concentration of secondary stabilizer (% *w*/*v*)	−1.68	0.02
		−1	0.05
		0	0.10
		+1	0.15
		+1.68	0.18
X_3_	Milling times (min)	−1.68	32.96
		−1	50
		0	75
		+1	100
		+1.68	117.04

**Table 2:** Observed response for the 20 experimental runs in central composite design.

Run	Independent Factors			Experiment Responses		
	X_1_/(% *w*/*v*)	X_2_/(% *w*/*v*)	X_3_/(min)	Particle Size (nm)	PDI	Zeta Potential (mV)
1	0.97	0.10	75.00	152.6	0.254	−20.3
2	0.80	0.02	75.00	342.6	0.351	−17.9
3	0.80	0.18	75.00	371.8	0.208	−33.0
4	0.63	0.10	75.00	171.9	0.204	−30.5
5	0.90	0.05	100.00	217.0	0.317	−19.9
6	0.80	0.10	117.04	160.9	0.190	−33.0
7	0.90	0.05	50.00	217.0	0.367	−22.4
8	0.90	0.15	50.00	252.0	0.224	−30.9
9	0.80	0.10	75.00	162.3	0.205	−34.6
10	0.90	0.15	100.00	248.0	0.194	−23.0
11	0.80	0.10	75.00	153.4	0.207	−34.3
12	0.80	0.10	75.00	156.9	0.217	−34.1
13	0.70	0.15	50.00	243.0	0.217	−35.9
14	0.80	0.10	75.00	158.7	0.210	−35.0
15	0.80	0.10	75.00	156.5	0.215	−34.8
16	0.80	0.10	32.96	176.4	0.255	−34.8
17	0.80	0.10	75.00	157.5	0.220	−34.6
18	0.70	0.05	100.00	248.5	0.232	−26.8
19	0.70	0.05	50.00	263.0	0.262	−20.8
20	0.70	0.15	100.00	224.1	0.201	−36.4

**Table 3:** Summary of the analysis of variance (ANOVA) for responses Y1, Y2 and Y3 in the quadratic model.

Source	Particle Size(Y_1_)				PDI (Y_2_)				Zeta Potential(Y_3_)			
	Sum of Squares	Degree of Freedom	F-Value	*p* Value (Prob > F)	Sum of Squares	Degree of Freedom	F-Value	*p* Value (Prob > F)	Sum of Squares	Degree of Freedom	F-Value	*p* Value (Prob > F)
Model	77,170.9	9	339.2	<0.0001	0.049	9	89.1	<0.0001	782.3	9	300.0	<0.0001
X_1_	434.8	1	17.2	0.0020	5.501 × 10^−3^	1	90.7	<0.0001	122.2	1	421.8	<0.0001
X_2_	366.0	1	14.4	0.0035	0.025	1	409.9	<0.0001	278.7	1	962.0	<0.0001
X_3_	294.9	1	11.6	0.0066	4.055 × 10^−3^	1	66.9	<0.0001	3.5	1	12.1	0.0059
X_1_ X_2_	1523.5	1	60.2	<0.0001	4.512 × 10^−3^	1	74.4	<0.0001	21.4	1	74.0	<0.0001
X_1_ X_3_	108.0	1	4.2	0.0656	1.445 × 10^−4^	1	2.3	0.1536	35.7	1	123.2	<0.0001
X_2_ X_3_	8.8	1	0.3	0.5678	1.445 × 10^−4^	1	2.3	0.1536	14.8	1	51.2	<0.0001
X_1_^2^	112.2	1	4.4	0.0613	7.465 × 10^−4^	1	12.3	0.0056	168.6	1	582.2	<0.0001
X_2_^2^	74,120.5	1	2932.1	<0.0001	9.044 × 10^−3^	1	149.2	<0.0001	166.9	1	576.2	<0.0001
X_3_^2^	368.0	1	14.5	0.0034	3.459 × 10^−4^	1	5.7	0.0380	2.4	1	8.6	0.0149
Residual	252.7	10			6.060 × 10^−4^	10			2.9	10		
Lack of Fit	210.1	5	4.9	0.0524	4.307 × 10^−4^	5	2.4	0.1732	2.3	5	4.4	0.0640
Pure Error	42.6	5			1.753 × 10^−4^	5			0.5	5		
Cor Total	77,492.4	19			0.049	19			785.2	19		

**Table 4:** Verification of central composite design for optimization of CXL-NC formulation.

Verification Trial	PVP VA64 (%, *w*/*v*)	SDS (%, *w*/*v*)	Milling Times (min)	Actual by Predicted	Particle Size (nm)	PDI	Zeta Potential (mV)
1 ^a^	0.75	0.11	90	Predicted	159.3	0.194	−35.8
				Actual	152.4 ± 1.4	0.192 ± 0.012	−34.4 ± 0.6
				Error	4.33	1.03	3.91
2	0.85	0.06	55	Predicted	196.2	0.304	−27.2
				Actual	191.7 ± 2.9	0.291 ± 0.025	−26.1 ± 1.0
				Error	2.29	4.28	4.04
3	0.70	0.14	75	Predicted	204.7	0.200	−36.9
				Actual	201.7 ± 3.7	0.209 ± 0.014	−36.8 ± 0.5
				Error	1.47	4.50	0.27

^a^ Final optimized formulation. Actual measure values are expressed as the mean ± SD (n = 3). PVP: polyvinylpyrrolidone; SDS: sodiumdodecyl sulfate.

**Table 5:** Pharmacokinetic parametersof optimized CLX-NC andPM after oral administration(mean ± SD, n = 6).

Parameters	Optimized CLX-NC	Physical Mixture Contain CLX
*C*max (μg/mL)	7.88 ± 0.72	2.70 ± 0.25
*T*max (h)	1.50 ± 0.32	2.83 ± 0.41
AUC_(0–t)_ (μg·h/mL)	54.90 ± 0.30	16.04 ± 3.36
AUC_(0–∞)_ (μg·h/mL)	66.75 ± 2.51	21.53 ± 3.02
*t*_1/2_ (h)	0.89 ± 0.18	0.87 ± 0.09
MRT (h)	1.37 ± 0.04	1.31 ± 0.13

## Supplementary Materials

The following are available online at https://www.mdpi.com/1999-4923/11/7/328/s1, Figure S1: Viscosity values of the suspension containing different polymer stabilizer. Figure S2: Effects of critical material attributes (CMAs) and critical process parameters (CPPs) on critical quality attributes (CQAs) of CLX-NC.CMAs and CPPs include (A) PVP VA64 concentration, (B) SDS concentration, (C) milling times, and (D) balls size. Figure S3: Perturbation plots showed the effects of factors X1 (A), X2 (B) and X3 (C) on the responses Y_1_ (a), Y_2_ (b) and Y_3_ (c). X_1_ is the factor of concentration of polymer stabilizer (% w/v), X_2_ is the factor of concentration of secondary stabilizer (% *w*/*v*), X_3_ is the factor of milling time (min), Y_1_ is the response ofparticle size (nm), Y_2_ is the response ofPDI, Y_3_ is the response of zeta potential (mV). Figure S4: Design space (yellow overlap region) of CLX-NC for the desired critical quality attributes after evaluation of process and formulations variables. Figure S5: Particle size distributions of (A) optimized CLX-NC andCLX-NC after six mouthsstorage at (B) 4 °C and (C) 25 °C. Table S1: Preliminary risk assessment matrix elucidating the impact of critical material attributes (CMAs) and critical process parameters (CPPs) on critical quality attributes (CQAs). Table S2: Fit summary for responses Y_1_, Y_2_ and Y_3_.
