# Supplementary material for: Enhanced Oral Bioavailability of Celecoxib Nanocrystalline Solid Dispersion based on Wet Media Milling Technique: Formulation, Optimization and In Vitro/In Vivo Evaluation
Source: Pharmaceutics. 2019 Jul 11;11(7):328. doi: 10.3390/pharmaceutics11070328 (PMC6680726; doi:10.3390/pharmaceutics11070328)
Supplement: Supplementary file 1 [file pharmaceutics-11-00328-s001.pdf]

Supplementary Materials: Enhanced Oral Bioavailability of Celecoxib Nanocrystalline Solid Dispersion based on Wet Media Milling Technique: Formulation, Optimization and In Vitro/In Vivo Evaluation

Zhuang Ding †, Lili Wang †, Yangyang Xing, Yanna Zhao, Zhengping Wang and Jun Han *

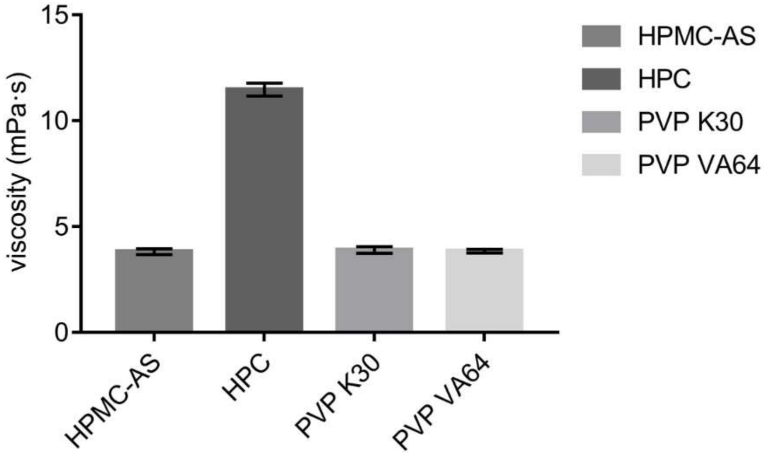

Figure 1. Viscosity values of the suspension containing different polymer stabilizer.

Experimental Method:

Viscosity Measurement

CLX (4% *w*/*v*), primary stabilizer (0.8% *w*/*v*) and secondary stabilizer (0.1% *w*/*v*) were dispersed in an aqueous solution (400 mL) using a magnetic stirrer operating at 500 rpm. PVP VA64, PVP K30, HPMC-AS and HPC were used singly as the primary stabilizer, while SDS was used as the secondary stabilizer. The viscosity of these suspensions were measured using a rotational Viscometer (DV-I + Digital, Brookfield, WI, USA) at 25 °C and 100 rpm speed by No. 1 rotor. Each sample was performed in triplicate.

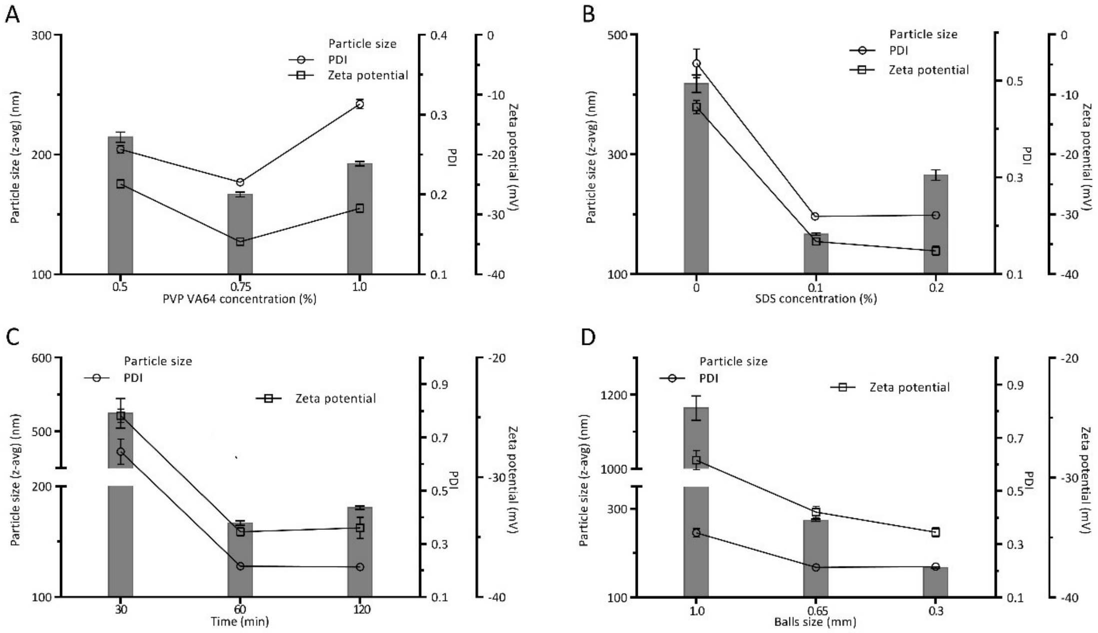

Figure 2. Effects of critical material attributes (CMAs) and critical process parameters (CPPs) on critical quality attributes (CQAs) of CLX-NC. CMAs and CPPs include (A) PVP VA64 concentration, (B) SDS concentration, (C) milling times, and (D) balls size.

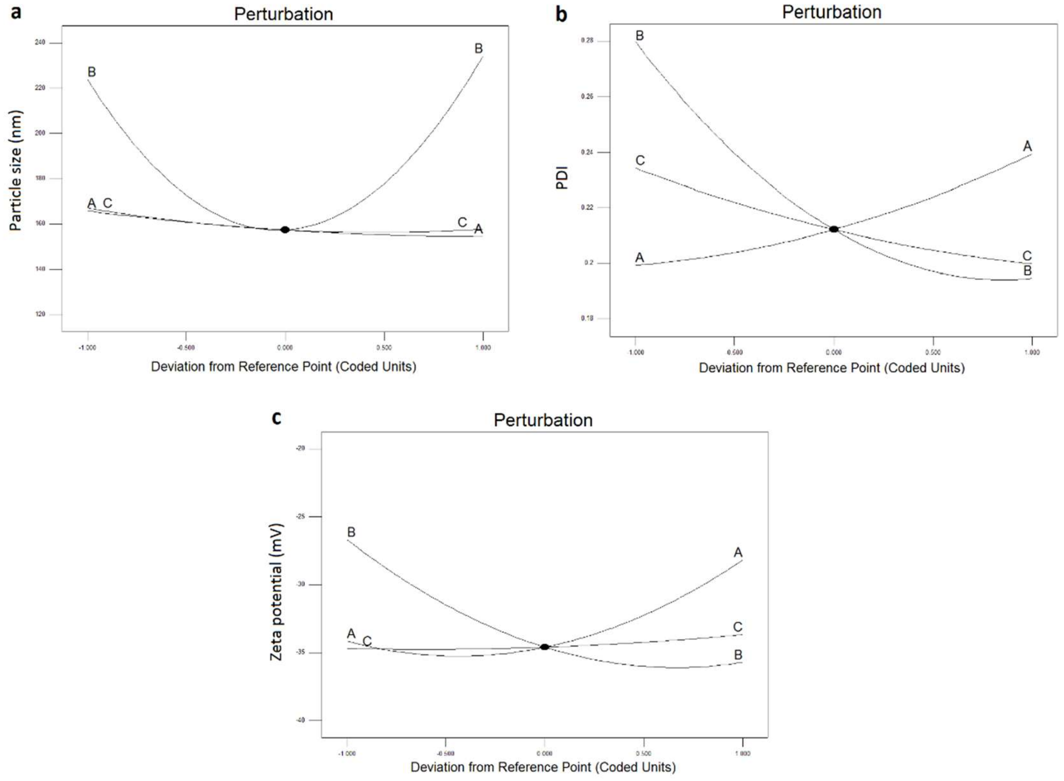

Figure 3. Perturbation plots showed the effects of factors X_1 (A), X_2 (B) and X_3 (C) on the responses Y_1 (a), Y_2 (b) and Y_3 (c). X_1 is the factor of concentration of polymer stabilizer (% w/v), X_2 is the factor of concentration of secondary stabilizer (% w/v), X_3 is the factor of milling time (min), Y_1 is the response of particle size (nm), Y_2 is the response of PDI, Y_3 is the response of zeta potential (mV).

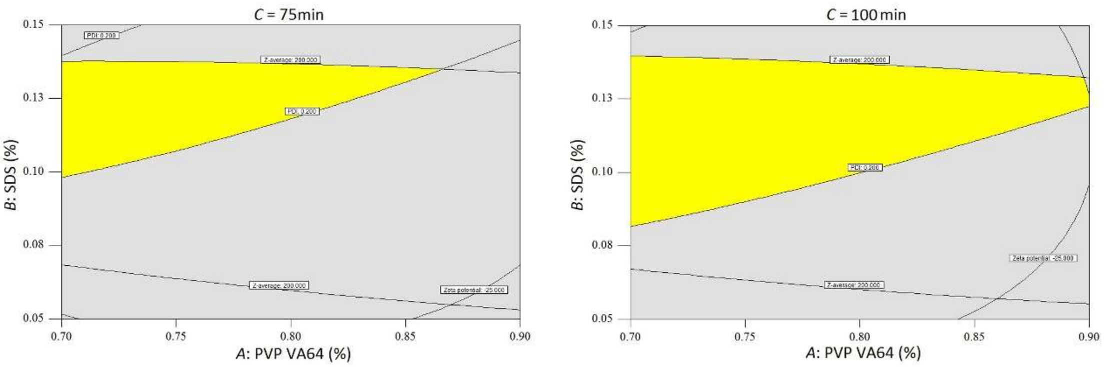

Figure 4. Design space (yellow overlap region) of CLX-NC for the desired critical quality attributes after evaluation of process and formulations variables.

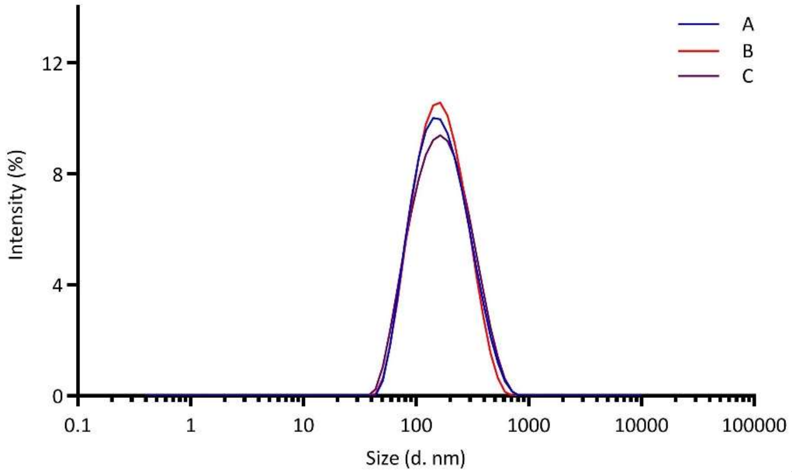

Figure 5. Particle size distributions of (A) optimized CLX-NC and CLX-NC after six mouths storage at (B) 4°C and (C) 25°C. (mean ± SD, n = 3).

Table 1. Preliminary risk assessment matrix elucidating the impact of critical material attributes (CMAs) and critical process parameters (CPPs) on critical quality attributes (CQAs).

CQAs	Risk Assessment Matrix						
	CMAs				CPPs		
	Primary stabilizer type	Primary stabilizer concentration (%)	Ionic surfactant concentration (%)	Drug amount [a] (mg)	Milling time (min)	Balls size (mm)	Mill speed [a] (rpm)
Particle size	High	High	High	Medium	High	High	High
PDI	High	High	High	Medium	High	Medium	Medium
Zeta potential	High	Medium	High	Medium	Medium	Medium	Medium

[a] Preliminary risk assessment was deduced based on the previous literatures [1,2].

Table 2. Fit summary for responses Y_1, Y_2 and Y_3.

	Sum of squares			df			F-value			p value (Prob>F)		
	Y_1	Y_2	Y_3	Y_1	Y_2	Y_3	Y_1	Y_2	Y_3	Y_1	Y_2	Y_3
Sequential model sum of squares												
Linear	1095.85	0.034	404.44	3	3	3	0.077	12.37	5.66	0.9717	0.0002	0.0077
2FI	1640.39	4.801E-003	72.00	3	3	3	0.095	2.07	1.01	0.9614	0.1532	0.4195
Quadratic	74434.69	9.425E-003	305.89	3	3	3	981.53	51.84	351.95	< 0.0001	< 0.0001	< 0.0001
Cubic	118.67	3.086E-004	0.033	4	4	4	1.33	1.56	0.017	0.3597	0.2988	0.9992
Residual	134.11	2.974E-004	2.86	6	6	6						
Total	9.738E+005	1.18	18367.68	20	20	20						

	R-squared			Adjusted R-squared			Predicted R-squared			PRESS			Std. Dev.		
	Y_1	Y_2	Y_3	Y_1	Y_2	Y_3	Y_1	Y_2	Y_3	Y_1	Y_2	Y_3	Y_1	Y_2	Y_3
Model summary statistics															
Linear	0.0142	0.6987	0.5151	-0.1707	0.6422	0.4241	-0.6322	0.5026	0.2416	1.264E+005	0.024	595.53	69.07	0.030	4.88
2FI	0.0353	0.7963	0.6068	-0.4099	0.7022	0.4253	-1.0642	0.4699	-0.0151	1.598E+005	0.026	797.06	75.80	0.028	4.87
Quadratic	0.9967	0.9877	0.9963	0.9938	0.9766	0.9930	0.9786	0.9284	0.9763	1653.26	3.525E-003	18.61	5.03	7.785E-003	0.54
Cubic	0.9983	0.9940	0.9964	0.9945	0.9809	0.9884	0.7388	0.4483	0.3446	20226.32	0.027	514.61	4.73	7.041E-003	0.69

References

1. Leena Peltonen, L. Design space and QbD approach for production of drug nanocrystals by wet media milling techniques. *Pharmaceutics* **2018**, *10*, 104.
2. Narayan, R.; Pednekar, A.; Bhuyan, D.; Gowda, C.; Koteshwara, K.B.; Nayak, U.Y. A top-down technique to improve the solubility and bioavailability of aceclofenac: In vitro and in vivo studies. *Int. J. Nanomed.* **2017**, *12*, 4921–4935.
